# Identification of Ovine *KRTAP28-1* and Its Association with Wool Fibre Diameter

**DOI:** 10.3390/ani9040142

**Published:** 2019-04-02

**Authors:** Lingrong Bai, Jing Wang, Huitong Zhou, Hua Gong, Jinzhong Tao, Jon G. H. Hickford

**Affiliations:** 1 Agricultural College, Ningxia University, Yinchuan 750021, China; lingrongbai@163.com; 2 College of Animal Science and Technology, Hebei North University, Zhangjiakou, Hebei 075131, China; wangjing197410@163.com; 3 Faculty of Agricultural and Life Sciences, Lincoln University, Lincoln 7647, New Zealand; zhouh@lincoln.ac.nz (H.Z.); hua.gong@lincoln.ac.nz (H.G.)

**Keywords:** sheep, keratin-associated protein KAP28-1 gene (*KRTAP28-1*), polymerase chain reaction (PCR), single-stranded conformational polymorphism (SSCP), variation, wool fibre diameter

## Abstract

**Simple Summary:**

Keratin-associated proteins (KAPs) are fundamental components of wool and hair fibres. They are split into three broad groups: the high sulphur (HS), the ultra-high sulphur (UHS) and the high glycine-tyrosine (HGT) KAPs. *KRTAP25-1* encodes a HS-KAP protein and the gene has recently been identified in humans. Here, we report the absence of a *KRTAP25-1* in sheep, and we describe a new HS-*KRTAP* (named *KRTAP28-1*) in the chromosome region where *KRTAP25-1* was expected to be found. Six variants (*A*−*F*) of *KRTAP28-1* containing eight single nucleotide polymorphisms (SNPs) and a TG dinucleotide repeat polymorphism were detected. One SNP was located upstream of the start codon and all the others were non-synonymous SNPs, including a nonsense SNP. The TG repeat polymorphism would lead to a reading frame shift at the carboxyl-terminal end. The association of *KRTAP28-1* with wool traits was investigated with 383 Southdown × Merino-cross lambs from seven sire lines. Of the four genotypes with a frequency over 5%, lambs of genotypes *AB* and *BD* produced wool of a smaller mean fibre diameter (MFD) than lambs of genotype *BC*. This shows that *KRTAP28-1* is associated with a key wool trait, and variation in this gene might therefore have value as a marker for improving that trait.

**Abstract:**

Keratin-associated proteins (KAPs) are a diverse group of proteins and form a matrix that cross-links keratin intermediate filaments in hair and wool fibres. From over 100 KAP genes (*KRTAPs*) identified in mammalian species, *KRTAP25-1* is a high sulphur (HS)-KAP gene, which has recently been described in humans. Here, we report the absence of *KRTAP25-1* in sheep, and describe a new HS-*KRTAP* (named *KRTAP28-1*) in the chromosome region where *KRTAP25-1* was expected to be found. Six variants (*A*−*F*) of *KRTAP28-1* containing eight single nucleotide polymorphisms (SNPs) and a TG repeat polymorphism were detected. One was positioned 30 bp upstream of the transcription start codon and all the others were non-synonymous SNPs, including a nonsense SNP. The TG repeat polymorphism would lead to a reading frame shift at the carboxyl-terminal end. The effect of *KRTAP28-1* on wool traits was investigated with 383 Southdown × Merino-cross lambs from seven sire lines. Of the four genotypes with a frequency of over 5%, lambs of genotypes *AB* and *BD* produced wool of a smaller MFD than lambs of genotype *BC*. This shows that *KRTAP28-1* is associated with wool fibre diameter, and that variation in this gene might have potential for use as a gene marker for reducing wool fibre diameter.

## 1. Introduction

Keratin-associated proteins (KAPs) are found in wool and hair fibres. They form a cross-linked network with the keratin intermediate filaments, and they are believed to play a central role in defining the physico-mechanical properties of these fibres. They have a high content of either cysteine, or both glycine and tyrosine, and traditionally they have been divided into three broad groups based on their amino acid composition: the high glycine–tyrosine (HGT; 35−60 mol% glycine and tyrosine), the high sulphur (HS; ≤30 mol% cysteine), and the ultra-high sulphur (UHS; >30 mol% cysteine) groups [1].

To date, over 100 KAP genes have been identified in mammalian species, including genes from sheep, rabbits, mice, and humans [1,2]. They have been positioned in 27 families (KAP1–KAP27), based on similarities in their encoded proteins, with each family comprising 1–12 members [1,3,4]. Bioinformatics analysis of the human genome sequence has led to the identification of 89 functional human *KRTAPs* and these have been placed into 25 KAP families: KAP1–KAP13, KAP15–KAP17 and KAP19–KAP27 [4,5].

The sequences of the *KRTAPs* appear to be conserved across species and based on sequence similarity; many of the human *KRTAP* orthologs have been identified in sheep and goats. These include ovine *KRTAP8-2* [6], ovine *KRTAP11-1* [7], ovine *KRTAP13-3* [8], ovine *KRTAP15-1* [9], ovine *KRTAP20-1* [10], ovine *KRTAP20-2* [11], ovine *KRTAP22-1* [12], ovine *KRTAP24-1* [13], ovine *KRTAP26-1* [14], caprine *KRTAP13-3* [15], caprine *KRTAP20-2* [16] and caprine *KRTAP24-1* [17]. Variation in *KRTAP26-1* has been recently reported to associate with a number of wool traits [14]. It would be interesting to identify other *KRTAPs* near *KRTAP26-1* and investigate whether they too are variable and how that variation, should it exist, affects wool traits.

*KRTAP25-1* is a HS-KAP gene that has been located in a position between *KRTAP24-1* and *KRTAP26-1* in humans [5]; but the gene has not been reported in any other species. In this study, we report our failed attempt to find a *KRTAP25-1* ortholog in the sheep genome, and report an apparently new *KRTAP* (named ovine *KRTAP28-1*) that was identified in a chromosomal region that should harbour *KRTAP25-1*, were it to exist. We report nucleotide sequence variation in the sequence identified, and its association with wool traits is also investigated.

## 2. Materials and Methods

This research involved animals and was undertaken in accordance with the Animal Welfare Act 1999 (New Zealand Government). The collection of blood drops from sheep by the nicking of their ears is covered by Section 7.5 Animal Identification, in: Code of Welfare: Sheep and Beef Cattle (2016). According to this Act, we are allowed to collect blood and wool as these are considered to be part of normal day-to-day farming activities.

### 2.1. Sheep Blood and Wool Samples

Three hundred and eighty-two Southdown × Merino-cross lambs from seven sire lines were investigated. The lambs were tagged with an identification number within 12 hours of birth. Their date of birth, weight at birth, birth rank (i.e., whether they were a single, twin or triplet), gender, and dam and sire were recorded. The ewes and lambs remained together until weaning. At tailing (lambs varied from 2–6 weeks of age), blood samples from all the lambs were collected onto TFN paper (Munktell Filter AB, Sweden), and genomic DNA was purified using the approach described by Zhou et al. [18].

Wool samples were collected at first shearing (12 months of age) from the mid-side of the lambs. Greasey fleece weight (GFW) was measured at shearing; and subsequently, mean fibre diameter (MFD), fibre diameter standard deviation (FDSD), coefficient of variation of fibre diameter (CVFD), mean staple length (MSL), mean fibre curvature (MFC), mean staple strength (MSS), prickle factor (PF) and wool yield (Yield) were measured using International Wool Textile Oganisation (IWTO) standardized methods by the New Zealand Wool Testing Authority Ltd (Ahuriri, Napier, NZ). Clean fleece weight (CFW) was then calculated from the GFW and Yield measurements.

### 2.2. Bioinformatic Analysis of the Sheep Genome Sequence

The human *KRTAP25-1* coding sequence (GenBank NM_001128598.1) was used to search the Ovine Genome Assembly Oar_v4.0 using the Basic Local Alignment Search Tool (BLAST) in order to ascertain whether there were any homologous sequences in the sheep genome. In humans, *KRTAP25-1* is located between *KRTAP24-1* and *KRTAP26-1* on chromosome 21 [5]. To further look for the presence of sequences homologous to human *KRTAP25-1*, the ovine genome sequence (approximately 37 kb in length) spanning *KRTAP24-1* and *KRTAP26-1* was retrieved from NCBI and specifically aligned with the human *KRTAP25-1* sequence. Open Reading Frame Finder (ORF Finder; www.ncbi.nlm.nih.gov/orffinder/) was used to detect any open reading frame (ORF) that could encode a cysteine-rich protein in the region between these two genes. 

### 2.3. PCR Amplification and Polymorphism Screening

The DNA sequences flanking the ORF detected above were utilised to design a pair of PCR primers to amplify a fragment containing the entire ORF. These primers were 5′-TAGACAAGCCATTCTCTGTTG-3′ and 5′-CATTCCAGTATTCCTGCCTG-3′, and they were synthesised by Integrated DNA Technologies (Coralville, IA, USA).

Amplification was performed in a 15-µL reaction containing the genomic DNA on one 1.2-mm punch of TFN paper, 150 µM of each deoxyribonucleotide triphosphate (dNTP; Bioline, London, UK), 2.5 mM of Mg^2+^, 0.25 µM of each primer, 0.5 U of *Taq* DNA polymerase (Qiagen, Hilden, Germany) and 1× reaction buffer supplied with the enzyme. The thermal profile consisted of an initial denaturation for 2 min at 94 °C, followed by 40 cycles of 30 s at 94 °C, 30 s at 58 °C and 40 s at 72 °C, concluding with a final extension incubation of 5 min at 72 °C. Amplification was carried out in S1000 thermal cyclers (Bio-Rad, Hercules, CA, USA).

The PCR amplicons were vetted for sequence variation by means of single-stranded conformational polymorphism (SSCP) analysis. Each amplicon (0.7 µL) was mixed with 7 µL of loading dye (98% formamide, 10 mM EDTA, 0.025% bromophenol blue, and 0.025% xylene cyanol). After denaturation at 90 °C for 5 min, the samples were promptly cooled on wet ice and then loaded on 16 cm × 18 cm, 10% acrylamide: bisacrylamide (37.5:1) (Bio-Rad) gels with 1% glycerol. Electrophoresis was performed using Protean II xi cells (Bio-Rad), at 320 V for 18 h at 14 °C in 0.5× Tris/Borate/EDTA (TBE) buffer. Gels were silver-stained according to the method of Byun et al. [19].

### 2.4. Sequencing of Variants and Sequence Analyses

PCR amplicons representing different banding patterns from sheep that seemed to be homozygous were sequenced in both directions by the Lincoln University (New Zealand) DNA sequencing service. Variants that were only found in sheep that seemed to be heterozygous were sequenced using an approach described by Gong et al. [20]. In this method, a band corresponding to the variant was removed as a gel sliver from the polyacrylamide gel, macerated and then used as a template for re-amplification with the original primers. This second amplicon was then sequenced.

Sequence translation, alignments and comparisons were undertaken using DNAMAN (version 5.2.10, Lynnon BioSoft, Vaudreuil, QC, Canada). A phylogenetic tree was constructed based on the predicted amino acid sequence of the ORF using MEGA version 7.0. The BLAST algorithm was used to search the NCBI GenBank (www.ncbi.nlm.nih.gov/) databases for homologous sequences.

### 2.5. Statistical Analyses

Hardy-Weinberg equilibrium (HWE) was calculated using POPGENE version 1.32 (Molecular Biology and Biotechnology Centre, University of Alberta, Edmonton, AB, Canada). 

Statistical analyses were undertaken using Minitab version 16 (Minitab Inc., State College, PA, USA). General Linear Mixed-Effect Models (GLMMs) were used to test the association between variation in *KRTAP28-1* and variation in ten wool traits. In these models, only sheep with genotypes that occurred in the population at a frequency over 5% were analysed using multiple pairwise comparisons with Bonferroni corrections. Sire and gender were found to affect all the wool traits (*p* < 0.05) and were thus incorporated into all the models as a random factor and a fixed factor, respectively. Birth rank was not found to affect wool traits and it was thus not incorporated into the models.

All *p*-values were considered statistically significant when *p* < 0.05 unless otherwise indicated. Trends were noted when 0.05 ≤ *p* < 0.1.

## 3. Results

### 3.1. The Absence of the KRTAP25-1 Homologue and the Presence of KRTAP28-1 in the Sheep Genome

**Figure 1:**
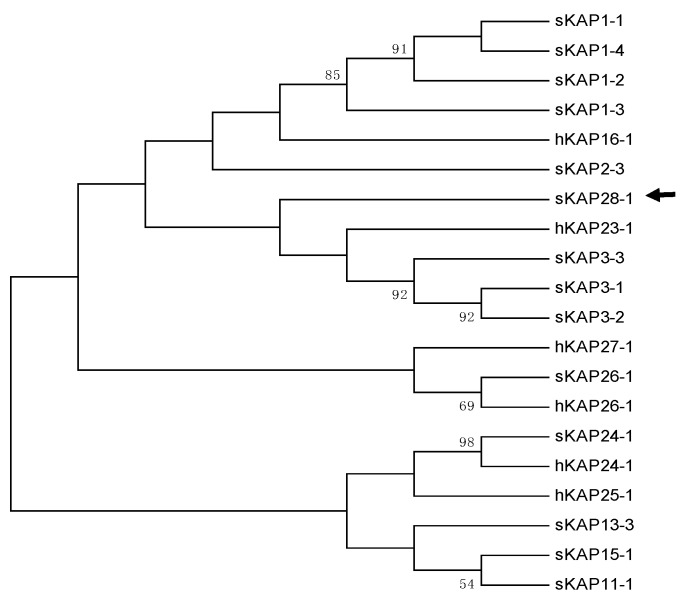
Phylogenetic tree for high sulphur keratin-associated proteins (HS-KAPs) that have been identified in sheep, along with human HS-KAPs. The tree is assembled using amino acid sequences. The sheep KAPs are specified with an “s” prefix, while the human KAPs are specified with an “h”. The numbers at the forks show the bootstrap confidence values, with only those equal to or greater than 50% being shown. The newly identified sheep KAP28-1 gene is marked with a horizontal arrow. The GenBank accession numbers for all other sheep KAPs are: NM_001159760 (sKAP1-1), HQ897975 (sKAP1-2), NM_001159761 (sKAP1-3), GQ507741 (sKAP1-4), P02443 (sKAP2-1), U60024 (sKAP2-3), M21099 (sKAP3-1), M21100 (sKAP3-2), M21103 (sKAP3-3), HQ595352 (sKAP11-1), JN377429 (sKAP13-3), KX817979 (sKAP15-1) and JX112014 (sKAP24-1). The accession numbers for the human KAPs are: NM_181623 (hKAP15-1), NM_001146182 (hKAP16-1), NM_181624 (hKAP23-1), NM_001085455 (hKAP24-1), NM_001128598 (hKAP25-1), NM_203405 (hKAP26-1) and NM_001077711 (hKAP27-1).

A BLAST search of the Ovine Genome Assembly Oar_v4.0 using the human *KRTAP25-1* coding sequence did not reveal any homologous sequence in the genome of the sheep. Alignment of the sheep genome sequence between *KRTAP24-1* and *KRTAP26-1* with human *KRTAP25-1* confirmed the apparent absence of a *KRTAP25-1* ortholog in sheep. However, bioinformatics analysis of this genome region identified a 408-bp ORF that would encode a protein containing 8.2 mol% cysteine. The most common amino acid residue found in this protein was serine (16.3 mol%). Evolutionary analysis revealed that this protein appeared to be different to human KAP25-1 and all other known HS-KAPs, and the most closely related KAPs were KAP3-n and KAP23-1 (Figure 1). Further sequence comparison with KAP3-n, KAP23-1 and KAP25-1 did not reveal any obvious sequence similarity for this protein (Supplementary Figure S1).

**Figure 2:**
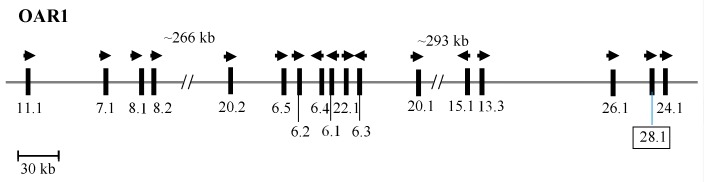
The KAP genes identified to date on sheep chromosome 1. The newly identified ovine *KRTAP28-1* is shown in a box. Vertical bars signify the location of the *KRTAPs* and the arrowheads specify the direction of transcription. The numbers below the bars are the names of the KAP genes (i.e., 11.1 is *KRTAP11-1*). The nucleotide distances are inexact and refer to NC_019458.2.

Taken together, this suggests that this ORF presents a newly identified HS-KAP gene, and one that would belong to a new family. It was therefore named SHEEP-*KRTAP28-1* according to the updated KAP nomenclature [2]. The chromosomal location of this newly identified *KRTAP28-1* sequence is displayed in Figure 2.

### 3.2. Variation in Ovine KRTAP28-1

**Figure 3:**
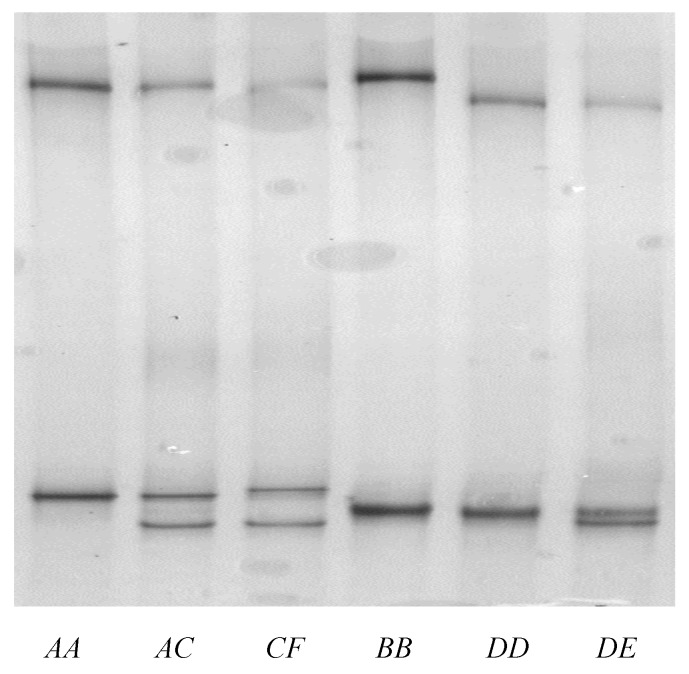
PCR-single-stranded conformational polymorphism of the ovine KAP28-1 gene. Five unique banding patterns corresponding to five variant sequences (*A*–*E*) are shown in either homozygous or heterozygous forms.

PCR-SSCP analysis revealed six different banding patterns for the notional ovine *KRTAP28-1*, with either one or a blend of two patterns being detected for each sheep (Figure 3). This is consistent with there being homozygous and heterozygous individuals, and it suggests that the amplicons were derived from a single gene.

**Figure 4:**
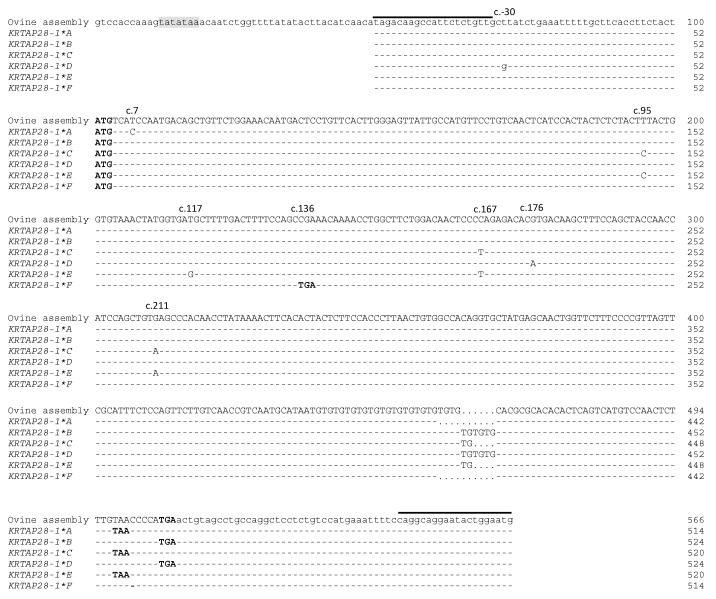
Alignment of the ovine *KRTAP28-1* variant sequences together with the ovine genome assembly sequence. Eight single nucleotide polymorphisms and a dinucleotide repeat polymorphism were detected in ovine *KRTAP28-1*. Nucleotides in the coding region are presented in upper case letters and those in the non-coding regions are in lower case. Dashes designate nucleotides indistinguishable to the ovine assembly, and dots have been introduced to increase the alignment. A putative TATA box is shaded, and the start and stop codons are presented in bold. The TG dinucleotide repeat region is boxed, and the horizontal lines designate the primer binding regions.

**Figure 5:**
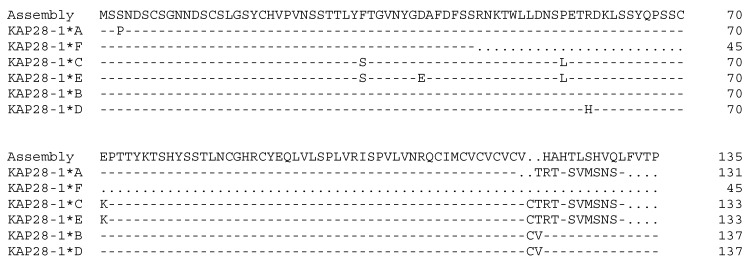
Predicted amino acid sequence alignment of the ovine *KRTAP28-1* variants, together with the ovine genome-derived assembly. Dashes signify amino acids identical to the assembly sequence, and dots have been introduced to increase the alignment.

The sequencing of PCR amplicons representative of these banding patterns revealed eight single nucleotide polymorphisms (SNPs) and a TG dinucleotide repeat (Figure 4). One SNP (c.-30T/G) was located upstream of the start codon, whereas all of the other SNPs were located in the coding region. These were c.7T/C, c.95T/C, c.117T/G, c.136C/T, c.167c/T, c.176G/A, and c.211G/A. All of the coding region SNPs were non-synonymous, and one of the non-synonymous SNPs could create a premature stop codon at position 46 of the putative polypeptide (Figure 5). There were 11 TG repeats found in variants *A* and *F*, 14 TG repeats in variants *C* and *E*, and 16 TG repeats in variants *B* and *D*. This TG dinucleotide repeat polymorphism would lead to a reading frame shift in the carboxyl terminus of the protein, and create a novel in-frame stop codon (Figure 5).

In total, ten different genotypes were observed (Table 1). The common genotypes were *AB*, *BB*, *BC* and *BD*, and the remaining six genotypes (*AA*, *AC*, *AD*, *BF*, *DD* and *DE*) were rare with a frequency of less than 5%. These gave frequencies of 14.9%, 57.5%, 9.1%, 16.4%, 1.8% and 0.3% for variants *A*, *B*, *C*, *D*, *E* and *F*, respectively. The genotype data were not in the Hardy–Weinberg Equilibrium for the sheep population investigated (Table 1). 

### 3.3. Effect of KRTAP28-1 on Wool Traits

As genotypes *AA*, *AC*, *AD*, *BF*, *DD* and *DE* were detected at a low frequency (less than 5%), the sheep with these genotypes were not included in the association analysis. Associations were therefore only tested for the sheep with the four genotypes: *AB*, *BB*, *BC* and *BD*.

The *KRTAP28-1* genotype was found to affect MFD. Sheep with genotypes *AB* and *BD* produced wool with smaller MFD than sheep of genotype *BC* (Table 2). No effects on other wool traits were detected. 

## 4. Discussion

This study reports the identification of what appears to be a new KAP gene in sheep. The low sequence similarity to all of the other known ovine HS-KAP genes, along with the high level of sequence variability between these genes, suggests that this novel gene should be assigned to a new family. The protein encoded by this new KAP gene would contain a lower than expected content of cysteine (8.2 mol%), compared to typical HS-KAPs, although a comparably low content of cysteine has been reported for *KRTAP24-1* [13] and *KRTAP26-1* [14]. This supports the assignment of this gene to the HS-KAP group. Given that up to 27 KAP families have been identified across mammalian species [2], this new KAP family was therefore named KAP28, and the gene was named *KRTAP28-1*. 

This putative ovine *KRTAP28-1* sequence is located between ovine *KRTAP26-1* and ovine *KRTAP24-1* on sheep chromosome 1, and in a corresponding region to where *KRTAP25-1* is found in this group of KAP genes in humans [5]. While it could therefore be argued that the ovine and human sequences are homologues, the sequence differences between human *KRTAP25-1* and the notional ovine *KRTAP28-1* would suggest otherwise (see Supplementary Figure S1).

The expression of *KRTAP25-1* has not been detected in Caucasians; but it remains a possibility that the expression may be individual or population-specific, with it perhaps being weakly expressed in Caucasians but strongly expressed in Orientals [21]. This would suggest that *KRTAP25-1* may contribute to racial differences in the human hair fibre. In this context, the apparent absence of *KRTAP25-1* and its substitution with *KRTAP28-1* in these sheep might possibly suggest that *KRTAP28-1* plays a role in defining a unique characteristic or breed-specific trait in wool fibres; but the occurrence of this gene in other breeds needs to be confirmed beforehand. 

The detection of six variant sequences for ovine *KRTAP28-1* is consistent with the observation that *KRTAPs* are polymorphic. There are, however, some unique characteristics of this variation. First is the presence of a dinucleotide repeat sequence variation in the coding region. While nucleotide repeat polymorphisms that do not result in reading frame shifts have been reported in other *KRTAPs*, including *KRTAP1-1* [22], *KRTAP5-4* [23], *KRTAP6-1* [24], and *KRTAP6-3* [25], a frame shift nucleotide repeat polymorphism has not been reported previously. The dinucleotide repeat polymorphism would potentially lead to changes in the carboxyl terminal sequence and the length of the protein. This may have a major effect on the structure and/or function of the protein, assuming all the variants are expressed. Second is the seemingly high frequency of occurrence of SNPs. The presence of eight SNPs in the 473–483 bp fragment amplified (excluding the primer-binding regions) would give a density of 16.6–16.9 SNPs per kb. This is more than three times the average density of 4.9 SNPs per kb across the sheep genome described by Kijas et al. [26]. This provides further evidence that *KRTAPs* have come about via unique evolutionary processes, including such things as gene conversion, as has been suggested for *KRTAP1-n* [22,27]. Third, is the predominance of non-synonymous SNPs. While SNPs are commonly found in *KRTAPs*, the nature of the SNPs observed in different *KRTAPs* appears to be different. In some *KRTAPs*, such as *KRTAP1-2* [28,29], *KRTAP6-2* [25], *KRTAP8-1* [30], *KRTAP11-1* [7] and *KRTAP26-1* [14], synonymous SNPs prevail, whereas in other *KRTAPs* such as *KRTAP1-4* [27], *KRTAP5-4* [23], *KRTAP6-5* [25] and *KRTAP13-1* [8], non-synonymous SNPs are found to predominate. Having a very high level of non-synonymous SNPs, as would appear to be the case with the putative *KRTAP28-1*, would appear to be quite unique. A high non-synonymous SNP rate would suggest that selective pressure may have been exerted on *KRTAP28-1*. Lastly is the unusual presence of a nonsense SNP. Nonsense SNPs are rare in *KRTAPs* and have only been reported for *KRTAP20-2* [11]. In *KRTAP20-2*, the nonsense SNP is located near the stop codon, while in *KRTAP28-1* the nonsense SNP is located early in the coding sequence and it would result in the loss of approximately two-thirds of the protein. If expressed, this would have a major effect on the structure and/or function of the protein and hence it would potentially have a major effect on fibre traits. However, the effect of this SNP on wool traits could not be investigated here due to a low frequency of the variant in the sheep investigated in this study. Further studies are required to investigate the effect of this SNP in a large sheep population, together with the expression of *KRTAP28-1* variants.

The deviations of genotypes from HWE may be due to a variety of causes, such as genotyping errors, outbreeding or inbreeding, sampling bias, natural selection, and sequence mutation/exchange. As the DNA samples were genotyped using PCR-SSCP, which is a reliable technique for determining variants, it is unlikely that the deviation from HWE for the *KRTAP28-1* variation is caused by genotyping errors. Outbreeding was not possible for these sheep, and inbreeding is unlikely to be a cause, as inbreeding might lead to increased homozygosity, which is not apparent given the diversity of the heterozygous genotypes that were observed. All of the progeny from the seven sire lines were investigated in this study, suggesting that sampling bias is unlikely to be a cause. Considering the functional significance of *KRTAP28-1*, natural selection is unlikely to be a cause as well. Given that *KRTAPs* undergo a high level of sequence variation [1] and DNA recombination events have been suggested for some *KRTAPs* [21,26], the deviation of the *KRTAP28-1* genotype data from HWE is possibly caused by DNA mutation and/or DNA recombination; but further investigations are required to confirm this.

The finding that wool of the *AB* and *BD* genotypes had a smaller MFD than wool of the *BC* genotype suggests that variation in *KRTAP28-1* is associated with MFD, and that *A* and *D* are associated with a decrease in MFD while *C* is associated with an increase in MFD. This association with *KRTAP28-1* is somewhat different to that reported for nearby *KRTAP26-1* [14]. Despite there being an association with MFD, variation in *KRTAP26-1* also affects FDSD, PF and MSL [14]. It would therefore seem unlikely that the effect detected for *KRTAP28-1* is due to the close linkage to *KRTAP26-1* on ovine chromosome 1. Given that fifteen other *KRTAPs* have been identified in a cluster with *KRTAP28-1* and *KRTAP26-1* [1,10,11,12,14], and that all of these *KRTAPs* are polymorphic and potentially expressed in the wool fibre, it is potentially very difficult to unravel the effects for individual *KRTAPs*. Nevertheless, the association detected in this study suggests that variation in *KRTAP28-1* should be further investigated with regards to its effect on wool fibre diameter.

## 5. Conclusions

This study identified a new KAP gene in sheep and this gene was found to belong to a previously identified KAP family. We reported variation in the gene and this variation was found to be associated with wool fibre diameter. These results may be useful in the development of gene markers for reducing wool fibre diameter.

## Figures and Tables

**Table 1:** Genotype count and Hardy–Weinberg Equilibrium (HWE) test for ovine *KRTAP28-1*.

Genotype	Observed	Expected	Frequency (%)
*AA*	7	8.4	1.8
*AB*	81	65.6	21.2
*AC*	14	10.5	3.7
*AD*	5	18.7	1.3
*BB*	103	126.0	27.0
*BC*	56	40.3	14.7
*BD*	94	71.9	24.6
*BF*	2	1.2	0.5
*DD*	6	10.2	1.6
*DE*	14	2.3	3.7
HWE	*p* < 0.001		

**Table 2:** Association of the *KRTAP28-1* genotype and wool traits.

Trait ^1^	Mean ± SE ^2^				*p* ^3^
	*AB* (*n* = 81)	*BB* (*n* = 103)	*BC* (*n* = 56)	*BD* (*n* = 94)	
GFW (kg)	2.39 ± 0.06	2.34 ± 0.06	2.44 ± 0.06	2.35 ± 0.05	0.578
CFW (kg)	1.75 ± 0.05	1.71 ± 0.05	1.74 ± 0.05	1.73 ± 0.04	0.893
Yield (%)	73.3 ± 0.93	72.8 ± 0.84	71.2 ± 0.96	73.2 ± 0.81	0.369
MSL (mm)	84.6 ± 1.70	85.3 ± 1.53	82.9 ± 1.76	85.6 ± 1.48	0.626
MSS (N/ktex)	21.7 ± 1.08	23.0 ± 0.97	24.5 ± 1.12	22.0 ± 0.94	0.328
MFD (µm)	19.0 ± 0.26 ^b^	19.4 ± 0.23 ^ab^	19.9 ± 0.27 ^a^	18.8 ± 0.23 ^b^	**0.019**
FDSD (µm)	4.12 ± 0.09	4.22 ± 0.08	4.30 ± 0.10	4.04 ± 0.08	0.180
CVFD (%)	21.7 ± 0.33	21.6 ± 0.30	21.5 ± 0.34	21.4 ± 0.29	0.748
MFC (^o^/mm)	86.6 ± 2.32	86.4 ± 2.09	91.8 ± 2.40	87.1 ± 2.02	0.279
PF (%)	2.00 ± 0.49	2.49 ± 0.44	2.97 ± 0.50	1.78 ± 0.42	0.340

^1^ GFW—Greasy Fleece Weight; CFW—Clean Fleece Weight; MFD—Mean Fibre Diameter; FDSD—Fibre Diameter Standard Deviation; CVFD—Coefficient of Variation of Fibre Diameter; MSL—Mean Staple Length; MSS—Mean Staple Strength; MFC—Mean Fibre Curvature; PF—Prickle Factor (percentage of fibres over 30 µm). ^2^ Estimated marginal means, standard errors of those means and *p*-values derived from General Linear Mixed-Effect Models (GLMMs). Means within rows that do not share a superscript letter (e.g., a,b) were different at *p* < 0.05. ^3^ *p*-values < 0.05 are in bold.

## Supplementary Materials

The following are available online at https://www.mdpi.com/2076-2615/9/4/142/s1, Figure S1. Comparison of the predicated amino acid sequences of the newly identified ovine *KAP28-1* gene and other similar KAP genes.
