# Supplementary material for: Identification of Ovine KRTAP28-1 and Its Association with Wool Fibre Diameter
Source: Animals (Basel). 2019 Apr 2;9(4):142. doi: 10.3390/ani9040142 (PMC6523819; doi:10.3390/ani9040142)
Supplement: Supplementary file 1 [file animals-09-00142-s001.pdf]

Supplementary Files: Identification of Ovine *KRTAP28-1* and Its Association with Wool Fibre Diameter

(A)

```
hKAP23-1    .MSYNCCCGNFSSHSCEGYLCYSGYSRGGSSYPSN..LVYSTEPLISQHL    47
sKAP28-1    .MSSNDSCSGNNDSCSLGSYCHVPVNSSTTLYFTG..VNYGDAFDFSSRN    47
sKAP3-1     ............MACCAPRCCSVRTGPATTICSSDKFCRCGVCLPSTCPH    38
sKAP3-2     ............MACCAPRCCSVRTGPATTICSSDKFCRCGVCLPSTCPH    38
sKAP3-3     ............MACCARLCCSVPTTPATTICSSDKFCRCGVCLPSTCPH    38
hKAP25-1    MHNRSQGFFFSSCHPQNHVSYGCQSPSFIFCRCQSLNFVSRTCYPLSYFS    50

hKAP23-1    PAGFLSLQGLSGDLLGNP................................    65
sKAP28-1    KTWLLDNSPETRDKLSSYQPSSCEPTTYKTSHYSSTLNCGHRCYEQLVLS    97
sKAP3-1     NISLLQPTCCDNSPVPCVYPDTYVPTCFLLNSSHPTPGLSGINLTTFIQP    88
sKAP3-2     DISLLQPTCCDNSPVPCYVPDTYVPTCFLLNSSHPTPGLSGINLTTFIQP    88
sKAP3-3     TVWFLEPTCCDNRPPPCHIPQPSVPTCFLLNSSQPTPGLESINLTTYTQP    88
hKAP25-1    YGNQTIGSISNSFRSLNYVSHSFQPISFMHSSFQPACSDFVGWQSPFLRR   100

hKAP23-1    ......................................                65
sKAP28-1    PLVRISPVLVNRQCIMCVCVCVCVHAHTLSHVQLFVTP               135
sKAP3-1     GCENVCEPRC............................                98
sKAP3-2     GCENVCEPRC............................                98
sKAP3-3     SCEPCIPSCC............................                98
hKAP25-1    TC....................................               102
```

(B)

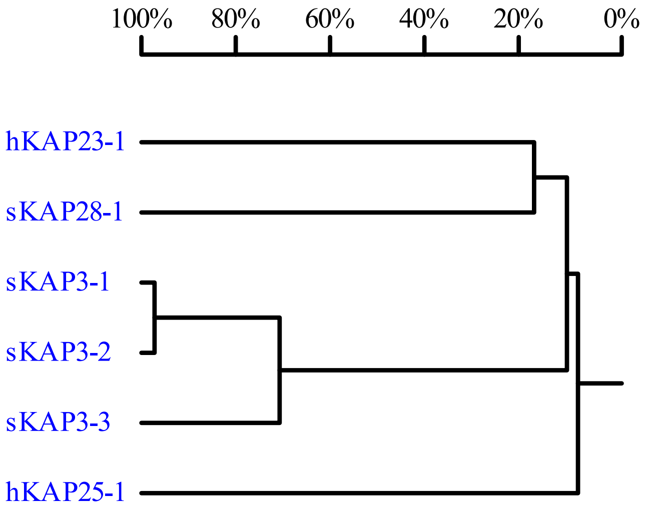

Figure S1. Comparison of the predicated amino acid sequences of the newly identified ovine KAP28-1 gene and other similar KAP genes. Three ovine KAP3-n genes and the human KAP23-1 gene that are shown to be most related to ovine KAP28-1 in Figure 1 are used for the comparison, together with the human KAP25-1 gene. The alignment of these sequences is shown (**A**), together with the sequence homology tree (**B**). The ovine KAPs are indicated with a prefix "s", while the human KAPs are indicated with "h". The GenBank accession numbers for other KAP genes are: M21099 (sKAP3-1), M21100 (sKAP3-2), M21103 (sKAP3-3), NM_181624 (hKAP23-1) and NM_001128598 (hKAP25-1).
